# A case report and systematic review of periorbital emphysema following nose blowing or sneezing

**DOI:** 10.1308/rcsann.2024.0090

**Published:** 2024-11-21

**Authors:** S Salar, O Edafe

**Affiliations:** ^1^ Leeds Teaching Hospitals NHS Trust, UK; ^2^ University of Sheffield, UK

**Keywords:** Subcutaneous emphysema, Nose blowing, Sneezing

## Abstract

Periorbital emphysema following nose blowing or sneezing is rare. Although it is often self-limiting, air trapping in the orbit can raise the intraocular pressure leading to visual complications. At present, the literature on this topic is confined to case reports. In this article, we present a rare case of periorbital emphysema following nose blowing in a 34-year-old woman and a subsequent systematic review that included 43 case reports of periorbital emphysema following similar mechanisms. Orbital wall fracture was seen in 70% and a defect in the lamina papyracea is the most common finding on computed tomography imaging. Surgical intervention was performed in 30% of patients; indications included visual compromise, paranasal osteoma and inflammatory disease. Most patients can be safely discharged on the same day with oral antibiotics unless there are clinical signs of visual compromise or sinonasal mass that may necessitate surgical intervention. The recurrence rate is low (3%) and almost all will resolve within 4 weeks.

## Background

Orbital emphysema is characterised by trapping of air in subcutaneous soft tissue around the orbit. This usually occurs following facial trauma or surgery.^1–3^ Periorbital emphysema without a history of recent facial trauma or surgery is rare; it has been reported mainly following nose blowing or sneezing. Other rare mechanisms include weightlifting and air travel.^4,5^ Typically, it presents as swelling surrounding the orbit, discomfort and erythema. Examination may reveal palpable crepitations in the early stage. This can eventually develop into orbital compartment syndrome compromising the optic nerve, and subsequently vision loss.^6^ An important differential diagnosis to consider is orbital cellulitis because it can progress to abscess formation and intracranial sepsis if left untreated.

We present a rare case of periorbital emphysema following nose blowing to further add another data point to the literature and describe our management. This clinical presentation formed the basis of a systematic review of the literature to identify reported cases. The evidence base for periorbital emphysema following nose blowing or sneezing is limited to case reports, given the rarity; there is a lack of summary data that characterise this condition. The aim of the systematic review was to undertake a quantitative summary of patient-level data and, using this, to characterise the clinical presentation, management strategies and outcomes.

## Case history

A 34-year-old woman was referred acutely to the ear, nose and throat department with a history of left periorbital swelling following nose blowing. She initially presented to the emergency department within 2h of developing sudden swelling around her left eye with some associated discomfort. There was no preceding sinonasal symptoms, no trauma or history of sinonasal or orbital surgery. There was no significant past medical history.

**Figure 1:**
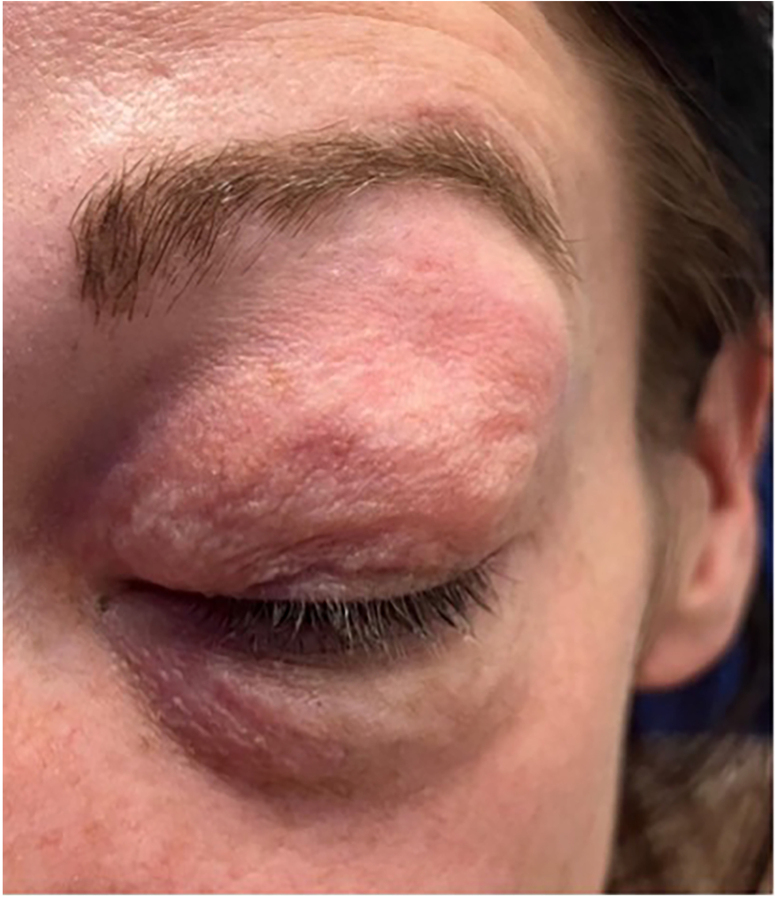
Left-sided periorbital emphysema

On clinical assessment, she had normal clinical observations, the left eyelid was closed due to swelling, and there was crepitus of the upper and lower eyelids (Figure 1). There was symmetrical visual acuity and normal eye examination. Nasoendoscopy showed a slight nasal septal deviation to the left and blood-stained secretions around the posterior aspect of the middle turbinate.

A computed tomography (CT) scan of the orbits and paranasal sinus showed the following: significant surgical emphysema in the superficial soft tissue of the left orbit, a fracture of the left lamina papyracea, and mucosal thickening of the ethmoid cells bilaterally, left frontal and sphenoid sinus.

She was discharged with a prophylactic course of oral amoxicillin/clavulanic 625mg three times daily for 7 days and advised to avoid nose blowing and sneezing until complete resolution of the swelling. The periorbital swelling resolved 6 days following initial presentation to the emergency department and there was no recurrence at follow-up.

## Methods

A systematic review of the literature was conducted in a population of patients with periorbital emphysema following nose blowing or sneezing.

### Eligibility criteria

A systematic search of PubMed and EMBASE from inception was performed using the following search terms: (Orbital emphysema OR Surgical emphysema) AND (nose blowing OR sneezing OR trauma).

We included all original articles in the English language evaluating periorbital emphysema in paediatric and adult patients with no history of trauma or facial/sinonasal surgery. The inclusion criteria for the patient population were as follows: nose blowing or sneezing as preceding mechanism for periorbital emphysema. We excluded patients with direct trauma as a cause (any trauma or surgery within 3 weeks of presentation) or any other mechanisms.

**Figure 2:**
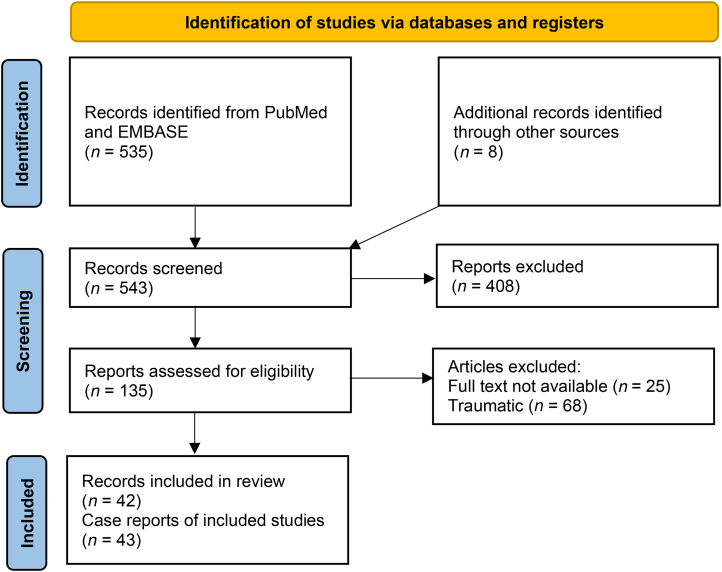
Prisma flow diagram to show the stages of screening with application of inclusion and exclusion criteria

Figure 2 shows the selection process of studies included in the systematic review. A total of 42 articles (43 patients) met the inclusion criteria.

### Data collection

Data on the following variables were collected using a standardised data collection tool on Microsoft Excel: age, gender, laterality, mechanism, recent trauma <3 weeks, past trauma >3 weeks, time from event to presentation, preceding sinonasal disease, visual acuity, pain, ophthalmoplegia, proptosis, raised intraocular pressure (IOP), relative afferent pupillary defect, imaging modality, evidence of fracture, presence of sinonasal disease on imaging, antibiotics (oral/intravenous), surgical intervention, inpatient/same-day discharge, time to resolution and recurrence. Where information on the variable was not available, the result was labelled ‘unknown’.

### Data analysis

The data were analysed using Microsoft Excel, producing descriptive statistics of patient demographics, clinical presentation, management approaches and outcomes. The data were presented in a table, with counts and percentages.

## Results

### Patient demographics

A total of 43 cases were included.^7–48^ The median (interquartile range) age at presentation was 35 (21–53) years. There were two cases in the paediatric population (<16 years of age).^10,14^ There were 29 males (29/43; 67%) and 14 females (14/43; 33%). All cases were unilateral (60% on the left eye and 40% on the right eye).

### Presentation

Periorbital emphysema occurred following nose blowing in 34/43 (79%) cases and after sneezing in 9/43 (21%). There were preceding sinonasal symptoms in 13/43 (30%) patients. Three patients had a preceding upper respiratory tract infection. Three patients had admitted to past medical history of facial trauma (range 4 months to 7 years).

### Symptoms

Table 1 shows the clinical presentation of the case. Visual acuity is represented as reduced if either explicitly documented as being reduced or reduced in comparison with the other eye. IOP was measured in 12 patients, of whom 4 had a raised IOP ranging between 25 and 80mmHg.

**Table 1:** Number (%) of cases with ophthalmology symptoms and signs

	Yes (%)	No (%)	Unknown (%)
Pain	22 (51)	21 (49)	0
Reduce visual acuity	7 (16)	20 (70)	6 (14)
Ophthalmoplegia	16 (37)	21 (49)	6 (14)
Proptosis	22 (51)	16 (37)	5 (12)
Raised intraocular pressure	4 (9)	8 (19)	31 (72)
Relative afferent pupillary defect	0	9 (21)	34 (79)

### Investigations

A CT scan was performed in the majority 40/43 (93%) of patients. Of these, 28/40 (70%) had an identifiable fracture: 10 (36%) involving the orbital floor and 18 (64%) involving the medial wall/lamina papyracea. Evidence of sinonasal disease on CT was found in 13/40 (33%) patients. Five were found to have a mass/lesion: one maxillary sinus polyp, three patients with ethmoidal osteoma and one with frontal osteoma.^15,22,25,28,47^

### Management

Initial surgical intervention was performed in 13/43 (30%) patients: 3 (23%) needle decompression; 3 (23%) excision of a mass lesion; 2 (15%) functional endoscopic sinus surgery; 2 (15%) repair of fracture; 1 lateral canthotomy and cantholysis, initially followed by endoscopic excision of middle turbinate and uncinate process 8h later; 1 frontal trephine; and 1 article did not specify the surgical intervention performed.^13–15,23,25,31,36,38,42–44,47^

Of the five patients with a lesion detected on CT scan, three underwent immediate surgical excision and one had a delayed surgical excision after two recurrent episodes of periorbital emphysema.^15,22,25,28,47^ Of the seven patients with sinusitis with or without abscess, four underwent endoscopic sinus surgery.^14,31,44^

At presentation, visual acuity was noted to be reduced in the affected eye in seven (16%) patients.^13,24,31,39,42–43,47^ All these patients were noted to have improvement in their visual acuity either following surgical decompression or at follow-up after a course of antibiotics. A total of four patients had raised IOP: two patients with raised IOP (37 and 80mmHg) and reduced visual acuity underwent surgical decompression.^31,43^ The other two patients were managed conservatively.^7,29^

Antibiotics were used in 30/43 (70%) patients, with predominantly oral preparations (77%). Of the cases, 26/43 (60%) were discharged on the same day.

### Follow-up

The time to complete resolution of the periorbital emphysema ranged from 7 to 28 days. Complete resolution of the emphysema was reported within 7 days of presentation in 15/33 (45%) patients, and 17/33 (52%) patients had complete resolution within 4 weeks. The time to resolution was unclear in ten patients.

One patient had a recurrence of their orbital emphysema.^28^ This patient had a CT confirming a right frontal sinus osteoma and was discharged the same day. Following this, the patient had two recurrent episodes of periorbital emphysema prior to surgical intervention with anterior orbitotomy and osteoma excision.

## Discussion

The predominant mechanism immediately preceding the onset of periorbital emphysema is nose blowing. This mechanism was explained by Gwaltney *et al*^49^ who identified higher mean maximal intranasal pressures of 66mmHg during nose blowing when compared with pressures of 4.6mmHg during sneezing.

Other potential non-direct trauma causes of periorbital emphysema have been reported. Monaghan *et al* described a case of a 21-year-old man who presented with periorbital emphysema following air travel.^4^ Of note, the patient reported a fall on his face two days prior but denied any nose blowing. He was seen before in the emergency department prior to the air travel and no fracture was suspected on assessment and on plain x-ray. Following the air travel, CT orbit was done which showed orbital floor fracture. In this case the changes in cabin pressure likely caused positive pressure of air into the sinus and through the defect of an initial occult fracture. Another case report described a patient with periorbital emphysema following weightlifting.^5^ There was no history of trauma in this patient. However, the case report did not comment on whether there was nose blowing or sneezing at any point. Interestingly, no fracture was seen on the CT scan with a 1.5mm slice, probably because of a false-negative result.

As in our case report, fracture of the lamina papyracea, the thinnest orbital wall, is the most common affected bone associated with nose blowing or sneezing. Orbital floor fractures also account for one-third of detected fractures. Orbital wall integrity may be affected by age, sinus disease and sinus surgery and where the wall is weakened or thin, possibly increasing the risk of fracture.^34,50^ In our case, there was no clinical history of chronic sinus disease; however, there was radiological evidence of chronic ethmoidal mucosal changes, a potential predisposing factor.

Across cases included in the systematic review, there was no history of recent trauma and only 30% had pre-existing history of sinonasal disease. Four patients (9%) had no visible fracture or evidence of concurrent sinonasal disease on CT scan.^8,18,21,24^ The absence of fracture in the cases may again be due to false negatives; three-dimensional CT has better sensitivity than plain axial CT in orbital fracture.^51^

Around one-third of patients underwent surgical intervention following presentation. The indications for surgery included visual compromise, associated paranasal mass and inflammatory sinus disease detected on imaging. This highlights the importance of urgent ophthalmology assessment at presentation and cross-sectional imaging in stratifying patients that may require surgical intervention.

Of note, two patients with reduce visual acuity did not have surgical intervention: the first patient had a visual acuity of 20/60 in the affected eye, whereas the second had a visual acuity recorded as 0.2 (with an acuity of 0.6 in the unaffected eye).^24,39^ The second patient also had minimally raised IOP of 25mmHg.^39^ The authors rationale for conservative management was not clearly stated. In addition, another patient with a raised intraocular pressure was managed conservatively; the pressure was recorded as 29mmHg but noted to resolve after 7h.^7^

Five cases were found to have an associated lesion on CT imaging.^15,22,25,28,47^ These were included in our review because they presented with periorbital emphysema after nose blowing and were not known to have any sinonasal lesions prior to presentation. There were three cases with ethmoidal osteoma penetrating through the medial orbital wall.^15,25,47^ These tumours caused a defect that allowed a route for air into the periorbital soft tissue. All cases required excision of the osteoma and repair of the medial orbital wall. There was one case in a 16-year-old boy with a frontal sinus osteoma extending through the orbital roof, who presented with periorbital swelling following nose blowing. Initially management was nonsurgical; however, the periorbital emphysema was recurrent, thus the osteoma was subsequently excised and the orbital roof repaired.^28^ Another case described periorbital emphysema following nose blowing with associated maxillary polyp; the author speculated this may have weakened the orbital wall.^22^ This case may represent an incidental finding and not be related to the fracture aetiology. The emphysema resolved after 15 days, and no surgery was performed.

Periorbital emphysema can compress the central retinal artery causing optic nerve ischaemia and eventually progress to permanent blindness unless the pressure is relieved. This typically manifests as reduced visual acuity and relative afferent pupillary defect and, so, it is important to undertake a thorough ophthalmology examination. Furthermore, not all patients who required surgical decompression had reduced visual acuity at the time of assessment. This highlights the importance of safety-netting and close monitoring for progression of emphysema. Antibiotics were commonly used across cases; this should be considered at presentation as a prophylaxis for periorbital infection.

The number of patients included in this review and potential confounding factors influencing treatment decision do not allow for inferential comparison to evaluate for associations between treatment modality (early needle decompression, prophylactic antibiotics) and outcomes. Indications for surgical intervention include orbital compartment syndrome, identified clinically by raised intraocular pressure and reduced visual acuity or associated localising lesion on imaging. There was no reported case of permanent visual loss. Across the cases, most patients with periorbital emphysema without associated sinonasal lesion were safely discharged on the same day without surgical intervention.

The pooled recurrence rate of periorbital emphysema was low. Cross-sectional imaging of the orbit and sinus can potentially identify cases with predisposing local pathology that will predispose to recurrence of emphysema. We also evaluated time to resolution across cases; it is commonly reported that most cases of orbital emphysema will resolve within 7 days, in keeping with our case presentation. However, in our systematic review, we found fewer than 50% of the cases completely resolved within 7 days, with the majority resolving within 4 weeks of presentation.

This is the first systematic review to summarise clinical cases of patients presenting with periorbital emphysema following nose blowing or sneezing. Although a rare presentation, our findings provided summary data for clinical characteristics, management and outcome for these patients. This finding will inform clinical consultations and aid decision making.

## Conclusion

Most cases of periorbital emphysema following nose blowing or sneezing are self-limiting and can be safely discharged on the same day with prophylactic antibiotics. A fracture of the lamina papyracea is the most common finding on CT. Cases with visual compromise or associated sinonasal mass may necessitate surgical intervention. Most cases will resolve within 4 weeks of presentation and the recurrence rate is low.
